# A longitudinal analysis of fatigue in colorectal cancer patients during chemotherapy

**DOI:** 10.1007/s00520-021-06097-w

**Published:** 2021-03-01

**Authors:** Xuemei Xian, Chenping Zhu, Yilin Chen, Binbin Huang, Didi Xu

**Affiliations:** Sir Run Run Shaw Hospital, School of Medicine, Zhejiang University, No. 3 East Qingchun Road, Hangzhou, 310016 China

**Keywords:** Colorectal cancer, Fatigue, Chemotherapy, Longitudinal design, CFS

## Abstract

**Purpose:**

The aim of this longitudinal study was to analyze trends in fatigue among colorectal cancer patients during chemotherapy and examine the predictors of multidimensional fatigue.

**Methods:**

A mixed sample of colorectal cancer patients who were receiving chemotherapy (*N* = 200) was recruited in China. The patients completed the Cancer Fatigue Scale (CFS) at baseline (before chemotherapy) and after 3 and 6 months of chemotherapy. Repeated measures ANOVAs were conducted to evaluate the effect of time on the CFS score. The data on violations of the statistical assumptions (independence, normality, and sphericity) from the repeated measures ANOVAs were examined. Stepwise regression analyses were conducted to evaluate the associations of the potential predictor variables at baseline on the total fatigue score and subscale scores at follow-up.

**Results:**

As chemotherapy progressed, significant increases in the three subscale scores and total scores were observed. Physical fatigue and total fatigue scores increased continuously during chemotherapy (*P* < 0.001). However, affective fatigue and cognitive fatigue scores increased significantly in the first 3 months (*P* < 0.001) and basically remained stable thereafter (*P* > 0.05). Multiple stepwise regression was used to analyze the predictors. The results showed that the baseline fatigue subscale score was the strongest predictor of each dimension of fatigue. In addition, age affected physical fatigue, and monthly income and education affected cognitive fatigue.

**Conclusion:**

Fatigue increased during chemotherapy. Early assessment and intervention may be better for controlling fatigue, especially in patients with higher baseline fatigue level, older age, and lower economic and educational levels.

## Introduction

Cancer-related fatigue is a common symptom in patients with colorectal cancer, especially in those receiving chemotherapy, with an incidence of up to 80% [1], and approximately 30% of patients experience persistent or chronic fatigue [2, 3]. The National Comprehensive Cancer Network defined cancer-related fatigue (CRF) as a “distressing persistent, subjective sense of physical, affective and/or cognitive tiredness or exhaustion related to cancer or cancer treatment that is not proportional to recent activity and interferes with usual functioning” [1]. CRF is a multidimensional symptom that includes three dimensions: physical fatigue, affective fatigue, and cognitive fatigue [1, 4].

Studies have shown that the pathogenesis of CRF may include physiological/biochemical, psychological, social, and other factors [3, 5–7], but the results are not completely consistent. Therefore, the management of CRF presents significant challenges for health professionals.

Fatigue has a profound effect on quality of life, for example, reducing patients’ energy levels, causing changes in daily life, and exacerbating other symptoms [8]. It may even reduce patients’ ability to engage in valuable life activities and socialize. Studies have shown that CRF affects patients longer and more severely than most other symptoms [9]. Greater knowledge of the factors that predict fatigue will help care providers better identify patients at risk for fatigue during treatment and help them develop the most effective individualized intervention plans. In addition, the adverse effects of fatigue on quality of life [8, 10], work capacity [11, 12], and compliance can be reduced through the provision of early intervention programs [13].

Previous longitudinal studies have revealed that patients who experience fatigue at the beginning of treatment are more likely to suffer from fatigue during chemotherapy [14, 15]. The other major predictive factors reported in the literature include depression [6, 15], age [11, 16], female sex [11], chemotherapy [11], occupational status [14], and physical limitations. However, most relevant studies have used the total fatigue score as a predictor and have not accounted for multidimensional fatigue. By definition, fatigue is multidimensional, affecting the body, emotion, and cognition, and there may be different factors influencing the characteristics of different dimensions of fatigue. Therefore, we consider it necessary to identify specific predictors of the physical, emotional, and cognitive dimensions of fatigue.

The purpose of this longitudinal study was to analyze trends in fatigue among colorectal cancer patients during chemotherapy and examine the predictors of multiple dimensions of fatigue. We estimated the predictive factors for severe fatigue based on the relationships between different baseline variables and fatigue over a 6-month chemotherapy period, but we did not identify the causes of fatigue. Referring to the multifactorial concept of the perpetuation of CRF, we tested the impact of sociodemographic, clinical, and baseline levels of fatigue on the total fatigue score and subscale scores.

## Methods

### Study design

A longitudinal study was conducted to assess the prevalence of fatigue among colorectal cancer patients before chemotherapy and after 3 and 6 months of chemotherapy and to examine the associations of the predictors with fatigue over a 6-month period.

### Patients and settings

The study took place between October 2017 and April 2018 at a teaching hospital in China. A group of 200 patients with colorectal cancer were recruited through convenience sampling. Patients were eligible if they (1) were more than 18 years old and had colorectal cancer, (2) had accepted postoperative chemotherapy, and (3) had agreed to participate in this study, had signed the informed consent form, had the ability to understand and write Chinese, and could communicate effectively with the researchers.

The exclusion criteria were as follows: patients who (1) had known psychiatric diseases or dementia, (2) had a history of malignancy or chemotherapy, (3) had received radiation concurrent with chemotherapy, (4) had immune disorders or were using immunosuppressive medication upon enrollment in the study, (5) had severe chronic metabolic diseases or nutritional disorders, and (6) had an estimated life expectancy of 6 months or less.

### Instruments

#### General information questionnaire

Demographic information and information on disease/treatment factors were collected from the patients’ medical records and via a study-specific patient information questionnaire that included items on age, sex, education level, income, employment status, marital status, type of chemotherapy (FOLFOX6 or XELOX), stage of the disease at diagnosis, and presence of colostomy.

#### Cancer Fatigue Scale

The Cancer Fatigue Scale was developed by Okuyama et al. [17]. This scale includes three subscales: physical fatigue, affective fatigue, and cognitive fatigue. There are a total of fifteen items on the scale. All items are scored on a 5-point Likert scale from 1 (“not at all”) to 5 (“extremely”). The physical subscale included 7 items (“easily tired,” “having urge to lie down,” “exhausted,” “heavy and tired,” “reluctant,” “fed-up,” and “don’t know what to do with yourself”). The affective subscale included 4 items (“energetic feeling,” “interest in something,” “encourage yourself to do something,” and “ability to concentrate”). The cognitive subscale included 4 items (“forgetful,” “errors while speaking,” “thinking has become slower,” and “careless”). Higher scores indicate a higher degree of fatigue. The test–retest reliability is 0.88, and the validity is good. In 2011, the CFS was translated into Chinese by Feng-ling Zhang et al. [18]. The Cronbach’s alpha of the Chinese version of the questionnaire is 0.86. We obtained permission to use the CFS-C from Zhang.

### Study procedures

This study was approved by the ethics committee of the Sir Run Run Shaw Hospital. Before chemotherapy, a clinical staff member explained the study to the patient and determined his/her willingness to participate. The research nurse met with the patients; determined their eligibility; provided them with written information on the study protocol, purpose, risks, and benefits and addressed their concerns; and obtained their written informed consent. Participation was completely voluntary for all patients. In general, chemotherapy for colorectal cancer usually occurs once per month for a total of 6 months. Therefore, after providing consent, the patients completed the questionnaires before chemotherapy and after 3 months (mid-chemotherapy) and 6 months (post-chemotherapy). All questionnaires were distributed and collected by two research nurses and completed by the patients anonymously with no interference. The research nurses met with the patients at the Clinical Research Center when they returned to the hospital for chemotherapy. To increase the accuracy of the questionnaires, the participants were given 15 min to complete them. All medical data were obtained from (electronic) patient records.

### Data analyses

All analyses were performed using IBM SPSS 21.0, with the statistical significance set at *P* < .05. Descriptive statistics are presented as the mean ± SD for numerical variables and *n* (%) for categorical variables. An independent sample *t*-test was performed to compare the differences in the continuous variables between the two groups when normality and homogeneity assumptions were satisfied; repeated measures ANOVAs were conducted to evaluate the effect of time on the CFS score. The data on violations of the statistical assumptions (independence, normality, and sphericity) from the repeated measures ANOVAs were examined. Stepwise regression analyses were conducted to evaluate the associations of the potential predictor variables at baseline with fatigue at follow-up.

## Results

### Sample characteristics

A total of 200 patients were eligible, agreed to participate in the study, and provided data, and 174 (87%) of these patients returned the questionnaire at all three measurement points. The demographic data for all the patients in the study are presented in Table 1. Among the 174 patients, 98 were male, and 76 were female. The mean age of all the patients was 60.53 years (SD 10.53). The majority of the patients had at least a middle school education (*n* = 154, 88.51%), were not working (*n* = 106, 60.92%), and had a monthly household income greater than 10,000¥ (*n* = 82, 47.3%). A total of 118 (67.82%) of the patients had stage III colorectal cancer and 34 (19.54%) had stage IV colorectal cancer. Among the patients with stage IV disease, 31 patients had liver metastases and 3 had lung metastases. Twenty-one patients with liver metastases underwent the resection of liver metastases concurrent with the primary resection, and 9 patients underwent radiofrequency ablation at the time of surgery. Two patients with lung metastases underwent radiofrequency ablation and then resection for the primary colorectal cancer. The other two patients only underwent the primary resection and did not receive treatment for the metastases. In total, 61.49% of the patients did not have a colostomy, and 51.72% received FOLFOX chemotherapy. The most common adverse reaction during chemotherapy was nausea and vomiting, with 27.58% of patients suffering from mild-to-moderate nausea and vomiting. A 5-HT3 receptor antagonist was used to prevent nausea and vomiting. Another common adverse event was myelosuppression, which occurred in 13.21% of the patients. Colony stimulating factor was used to treat myelosuppression. All patients completed 6 cycles of chemotherapy as planned. At the end of chemotherapy, 169 patients had no tumor recurrence, while 3 patients had new liver metastases. The 2 stage IV patients who did not receive treatment for their metastases achieved a partial response.

**Table 1:** Patient characteristics (*n* = 174)

Variables	*n* (%)
*Demographic characteristics*	
Age	
Mean (SD)	60.53(10.53)
Range	41–76
Sex	
Male	98(56.32)
Female	76(43.68)
Education	
Primary school	20(11.49)
Middle school	139(79.89)
University	15(8.62)
Marital status	
Married	145(83.33)
Divorced/separated/widowed	29(16.67)
Employment status	
Working	68(39.08)
Not working	106(60.92)
Monthly household income	
<5000 ¥	33(18.97)
5000–10,000 ¥	59(33.91)
>10,000 ¥	82(47.13)
*Medical characteristics*	
Diagnosis	
Colon cancer	87(50)
Rectal cancer	87(50)
Overall stage of disease	
Stage II	22(12.60)
Stage III	118(67.82)
Stage IV	34(19.54)
Colostomy	
Yes	67(38.51)
No	107(61.49)
Type of chemotherapy	
FOLFOX	90(51.72)
XELOX	84(48.28)

*Abbreviations: SD* standard deviation

### Prevalence of fatigue

**Fig. 1:**
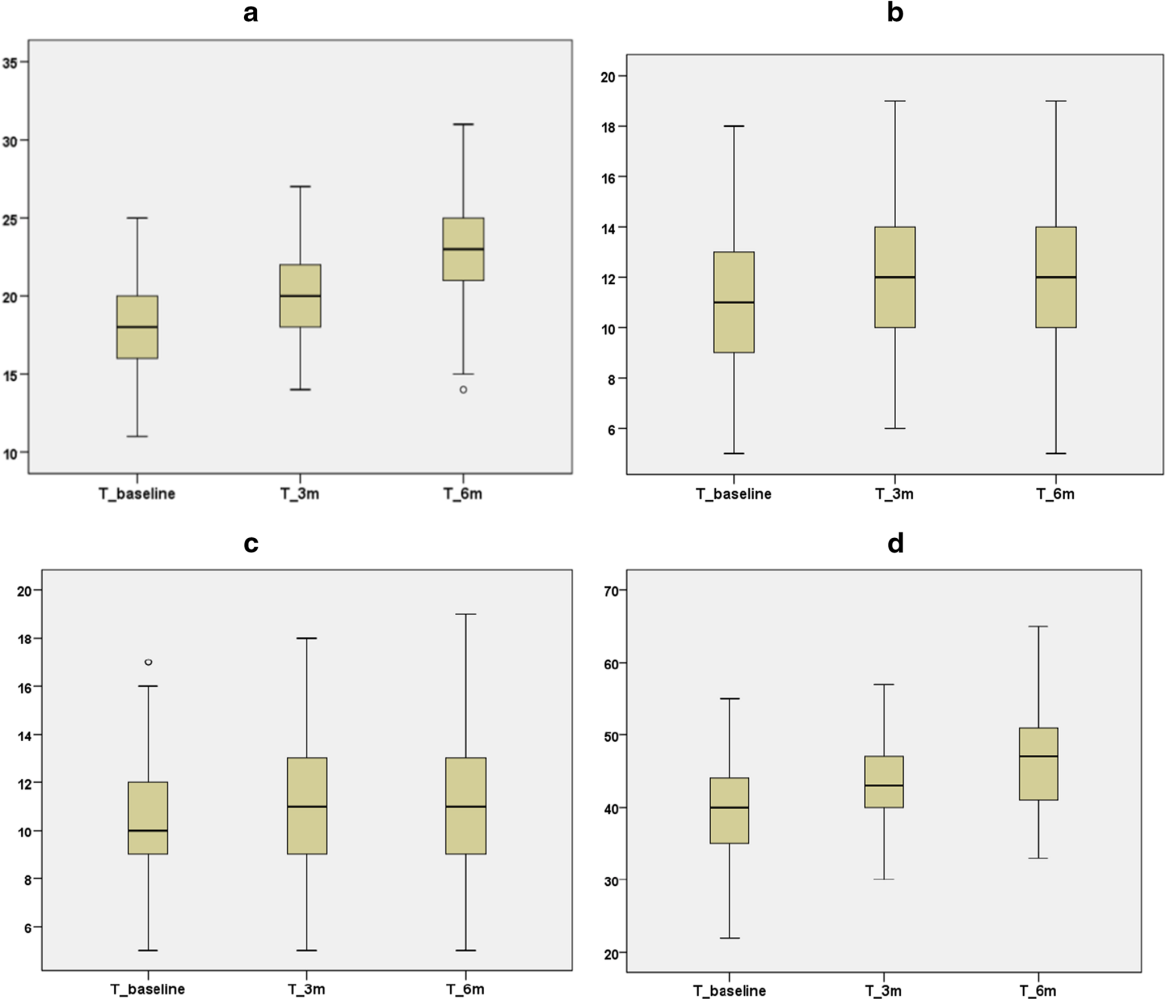
Trajectory of cancer-related fatigue during chemotherapy. **a** Physical fatigue. **b** Affective fatigue. **c** Cognitive fatigue. **d** Total fatigue

Table 2 shows that the mean fatigue scores were 18.5 (physical fatigue), 10.85 (emotional fatigue), and 10.58 (cognitive fatigue) at baseline. The corresponding follow-up values were 20.32, 12.02, and 11.07 at 3 months and 23.16, 12.01, and 11.25 at 6 months, respectively. As chemotherapy progressed, statistically significant increases in fatigue severity were observed in all three subscale scores and the total score (*P* < 0.01), which are shown in Fig. 1(a–d).

**Table 2:** Levels of fatigue at baseline and follow-up (*n* = 174)

	T_Baseline M(SD)	T_3 m M(SD)	T_6 m M(SD)	*F*	*P*
Physical fatigue	18.05(3.06)	20.32(2.84)	23.16(3.56)	171.37	<0.001
Affective fatigue	10.85(2.63)	12.02(2.56)	12.01(2.59)	20.72	<0.001
Cognitive fatigue	10.58(2.49)	11.07(2.39)	11.25(2.96)	4.57	0.01
Fatigue total score	39.48(6.48)	43.41(5.69)	46.42(6.63)	90.58	<0.001

*Abbreviations: SD* standard deviation

### Longitudinal differences in fatigue

The repeated measures ANOVAs showed statistically significant differences in all three subscale scores and the total score (Table 3). The physical fatigue score significantly increased between t_baseline and t_3 m (from 18.05 to 20.32 points; *p* < 0.001) as well as between t_3 m and t_6 m (from 20.32 to 23.16 points; *p* < 0.001). On the other hand, the affective fatigue and cognitive fatigue scores increased significantly between t_baseline and t_3 m (from 10.85 to 12.02, *p* < 0.001 and from 10.58 to 11.07, *p* < 0.001, respectively) and basically remained stable between t_3 m and t_6 m (from 12.02 to 12.01, *p* = 0.931, and from 11.07 to 11.25, *p* = 0.406, respectively). Finally, the total fatigue score increased continuously during chemotherapy, from 39.48 to 43.41 points (*p* < 0.001) between t_baseline and t_3 m and from 43.41 to 46.42 points (*p* < 0.001) between t_3 m and t_6 m.

**Table 3:** Differences in the subscale and total fatigue scores at all points of assessment (*n* = 174)

	T_3 m - T_baseline				T_6 m - T_3 m				*η*^2^
	Mean	SE	*p*	95% CI	Mean	SE	*p*	95% CI	
Physical fatigue	2.26	0.25	<0.001	1.77–2.76	2.84	0.27	<0.001	2.31–3.38	0.50
Affective fatigue	1.17	0.20	<0.001	0.78–1.56	−0.02	0.20	0.931	−0.41 to 0.38	0.11
Cognitive fatigue	0.49	0.21	0.021	0.07–0.90	0.18	0.22	0.406	−0.25 to 0.62	0.03
Total score	3.93	0.48	<0.001	2.99–4.87	3.01	0.48	<0.001	2.05–3.96	0.34

*Abbreviations: SE* standard error

### Factors associated with CRF during chemotherapy

Multiple stepwise regression was used to analyze the predictors. The introduced independent variables included demographic variables (age, sex, education, marital status, employment status, and monthly household income), disease characteristic variables (diagnosis, overall stage of disease, colostomy, and type of chemotherapy), the three baseline subscale scores (physical fatigue, affective fatigue, and cognitive fatigue), and the baseline total fatigue score. Table 4 shows that baseline fatigue subscale score was the strongest predictor of each dimension of fatigue. In addition, age had an effect on physical fatigue, and monthly income and education had effects on cognitive fatigue.

**Table 4:** Results of the multiple stepwise regression analysis of factors associated with cancer-related fatigue during chemotherapy among colorectal cancer patients (*n* = 174)

Dep. variables	Step	Indep. variables	*B*	Beta	*p*	95% CI	*R*^2^	Adj. *R*^2^	*P*
Physical fatigue	1	Physical (baseline)	0.32	0.28	<.001	(0.16, 0.49)	0.08	0.07	<.001
	2	Physical (baseline)	0.35	0.30	<.001	(0.18, 0.51)	0.12	0.11	<.001
		age	0.07	0.20	0.007	(0.02, 0.12)			
Affective fatigue	1	Affective (baseline)	0.33	0.33	<.001	(0.19, 0.47)	0.11	0.11	<.001
Cognitive fatigue	1	Cognitive (baseline)	0.29	0.24	0.001	(0.11, 0.46)	0.06	0.05	0.001
	2	Cognitive (baseline)	0.31	0.26	<.001	(0.14, 0.49)	0.10	0.09	<.001
		income	0.80	0.21	0.005	(0.24, 1.36)			
	3	Cognitive (baseline)	0.33	0.28	<.001	(0.16, 0.50)	0.13	0.11	<.001
		income	0.74	0.19	0.009	(0.18, 1.29)			
		Education	−0.76	−0.17	0.018	(−1.38, −0.13)			
Total score	1	Total (baseline)	0.77	0.30	<.001	(0.41, 1.13)	0.09	0.09	<.001
	2	Total (baseline)	0.81	0.32	<.001	(0.45, 1.16)	0.13	0.12	<.001
		Education	−1.82	−0.19	0.01	(−3.21, −0.44)			

## Discussion

The first goal of this study was to analyze time-varying trends in colorectal cancer fatigue in chemotherapy patients. We assessed the prevalence of fatigue among colorectal cancer patients who underwent chemotherapy, at baseline and at 3 and 6 months of chemotherapy. The results showed that fatigue was at a moderate level at baseline, with an average score of 39.48 (out of 75), and that it gradually increased with the progression of chemotherapy and reached a moderate-to-severe level at 6 months (46.42/75). It is difficult to compare fatigue across studies due to differences in the criteria used to define fatigue, the measurement instruments used, the time points selected, and the treatment modalities. Most previous studies have shown similar results: cancer patients receiving adjuvant chemotherapy have higher levels of fatigue than the general population, and their fatigue lasts for many years than that of the general population [6, 19–24].

In the present study, there were statistically significant, sustained increases in the physical fatigue and total fatigue scores. The affective fatigue and cognitive fatigue scores increased significantly between baseline and 3 months and remained almost stable between 3 and 6 months. The results of this study are similar to those of Kecke et al., who followed 354 women with cancer for 3 months and assessed their fatigue. They found no significant change in affective fatigue but increases in physical and cognitive fatigue [25].

Van et al., in a study of long-term cancer survivors, found that chemotherapy was associated with higher levels of fatigue [26]. Increased fatigue during chemotherapy may be associated with anemia, leukopenia, sleep disturbances, and other symptoms during chemotherapy, such as pain, gastrointestinal reactions, and bone marrow suppression [27]. However, other studies have shown inconsistent results; for example, Servaes et al. conducted a longitudinal study of breast cancer survivors and found no relationship between adjuvant therapy and fatigue [19]. In Muijen’s study, fatigue was surprisingly negatively correlated with chemotherapy, with nearly 75% of respondents who received chemotherapy reporting lower fatigue than those who did not receive chemotherapy [14]. In addition, Kuhnt Susanne found significant changes in fatigue in patients in a cancer rehabilitation program during 6 months of follow-up, and fatigue levels, particularly physical fatigue levels, were significantly lower than those at baseline [28]. These findings indicate that concurrent rehabilitation programs during chemotherapy may reduce fatigue.

Our second purpose was to examine the associations of the predictors with fatigue over a 6-month period. According to the results of the regression analyses, baseline fatigue was the strongest predictive factor of long-term fatigue during chemotherapy. Similar results have been reported in studies by Vardy et al., whose results indicated that the predictors of persistent fatigue are baseline fatigue, treatment, and emotional symptoms [6, 16, 29]. However, most of these studies followed the fatigue of cancer survivors, using fatigue at the end of acute treatment as a baseline for predicting long-term fatigue. Our study was a longitudinal study of fatigue during chemotherapy and used the fatigue score before chemotherapy as the baseline, which could better predict changes in fatigue during chemotherapy.

Although the proportion of variance explained by sociodemographic and clinical variables was small, the changes in the *R*^2^ value were significant. Age appeared to have a significant impact on physical fatigue. The decline in function in older patients may be an important reason for their more severe fatigue than that of younger patients. In other studies, age has also been shown to be an important and related risk factor, with the effect of age on long-term fatigue increasing as the disease progresses [30–33]. Thus, it seems that we should pay more attention to the fatigue of elderly cancer patients.

This study also found that income and education predicted cognitive fatigue, but to a lesser level than did other sociodemographic and clinical variables. Previous studies have focused less on the effects of income and education on fatigue, possibly because these studies rarely performed multidimensional analyses of fatigue. Patients with lower incomes and education levels may be more worried about economic problems, leading to the aggravation of their cognitive fatigue. In Muijen’s follow-up of cancer survivors, fatigue had a greater impact on the ability to work for lower-income cancer survivors [14]. This finding indicates that we should pay more attention to the fatigue of low-income cancer patients and consider reducing their financial burden through better health care plans. Meanwhile, in addition to being related to baseline fatigue, the total fatigue score was also related to education, which was also a predictor of cognitive fatigue. It seems that cognitive fatigue has a greater influence on the total fatigue score than the other two dimensions of fatigue, which needs to be confirmed by further research.

Although a baseline assessment was included in this study, other chronic conditions or other treatments may have an impact on patients’ fatigue during chemotherapy. In addition, CRF may persist for some time after chemotherapy. However, we assessed fatigue only before chemotherapy and at 3 months and 6 months after the beginning of chemotherapy, which may not have fully explained the trends in fatigue.

## Clinical implications

The results confirmed that baseline fatigue is an important predictor of fatigue during chemotherapy. There are different predictors of physical fatigue, emotional fatigue, and cognitive fatigue, so it is necessary to study the multiple dimensions of fatigue. Our results support the hypothesis that the evaluation of fatigue by caregivers as early as possible and early intervention for patients with higher baseline fatigue, older age, lower family income, and lower education may yield greater benefits for patients during chemotherapy.

## Data Availability

The authors had full control of all of the primary data and will allow the journal to review the data if requested to do so.
